# Successful airway management using a MultiViewScope handle with a stylet scope in a patient with Schwartz–Jampel syndrome

**DOI:** 10.1186/s40981-016-0062-5

**Published:** 2016-11-14

**Authors:** Keika Mukaihara, Kohei Godai, Tomotsugu Yamada, Maiko Hasegawa-Moriyama, Yuichi Kanmura

**Affiliations:** Department of Anesthesiology and Critical Care Medicine, Graduate School of Medical and Dental Sciences, Kagoshima University, 8-35-1 Sakuragaoka, Kagoshima, 890-8520 Japan

**Keywords:** MultiViewScope, Schwartz–Jampel syndrome, Stylet scope

## Abstract

Schwartz–Jampel syndrome (SJS) is a rare disorder characterized by micrognathia, kyphoscoliosis, and myotonia. The greatest challenge in the anesthetic management of patients with SJS is performing tracheal intubation. The MultiViewScope (MVS) is a video laryngoscope system in which the video monitor handle can be attached to a stylet scope, laryngoscope blade, or fiberscope. We report a 21-month-old boy with SJS who required general anesthesia. Direct laryngoscopy was impossible because of his limited mouth opening; however, his trachea was easily intubated using an MVS handle with a stylet scope. The MVS is useful for managing difficult airways associated with SJS.

**Electronic supplementary material:**

The online version of this article (doi:10.1186/s40981-016-0062-5) contains supplementary material, which is available to authorized users.

## Background

**Fig. 1:**
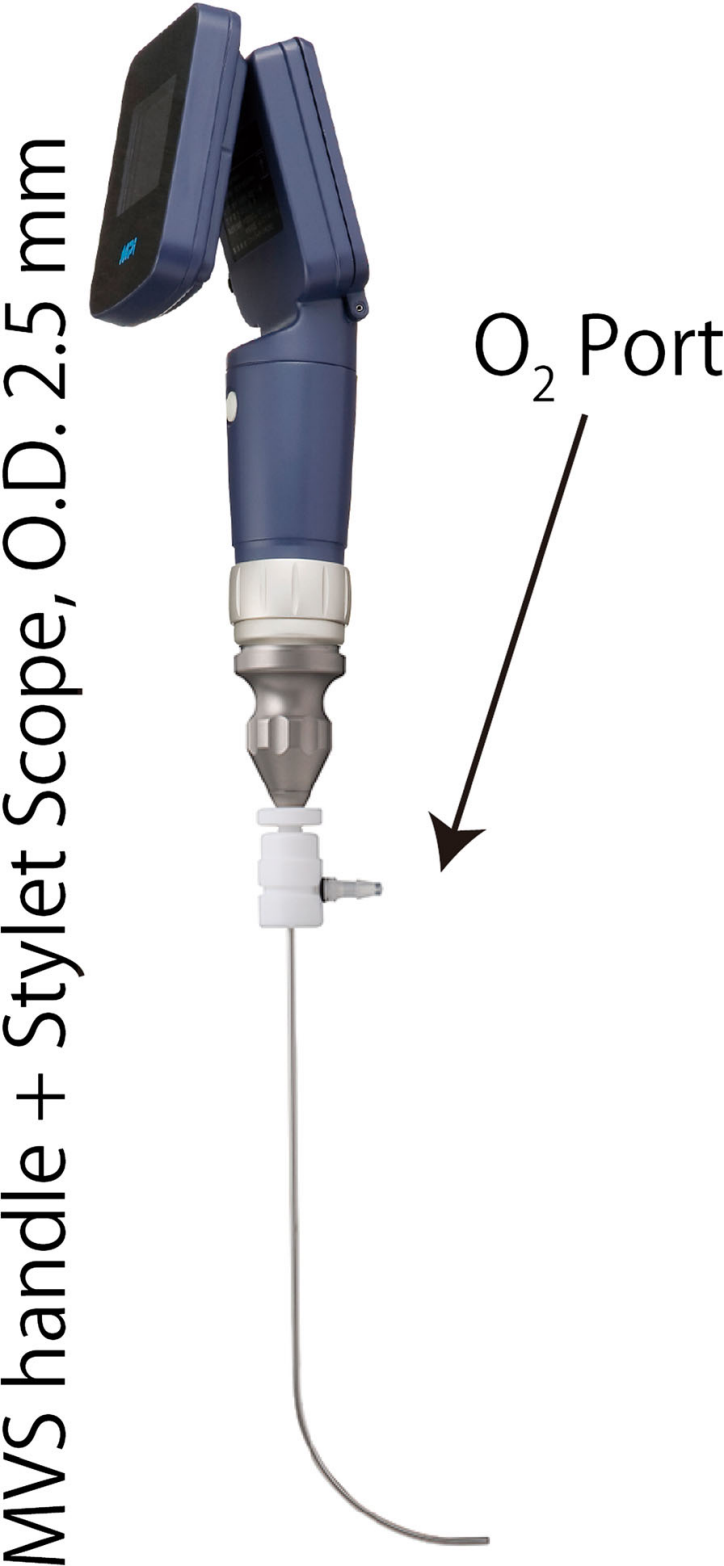
MultiViewScope (MVS) system. The stylet scope was attached to the video monitor handle

Schwartz–Jampel syndrome (SJS) is a rare disorder that is characterized clinically by micrognathia, short stature, kyphoscoliosis, and myotonia [1]. The greatest challenge in the anesthetic management of patients with SJS is performing tracheal intubation [2]. The MultiViewScope (MVS) (MPI, Tokyo, Japan) is a video laryngoscope system, in which the video monitor handle can be attached to a stylet scope, Miller blade, Macintosh blade, or fiberscope (Fig. 1). We describe a case in which the airway was successfully managed by using an MVS handle with a stylet scope.

## Case presentation

A 21-month-old boy (height, 74 cm; weight, 9.6 kg) was scheduled for inguinal hernia repair. He had been diagnosed with SJS because of his short stature, myotonia, physical appearance, and electromyographic pattern.

**Fig. 2:**
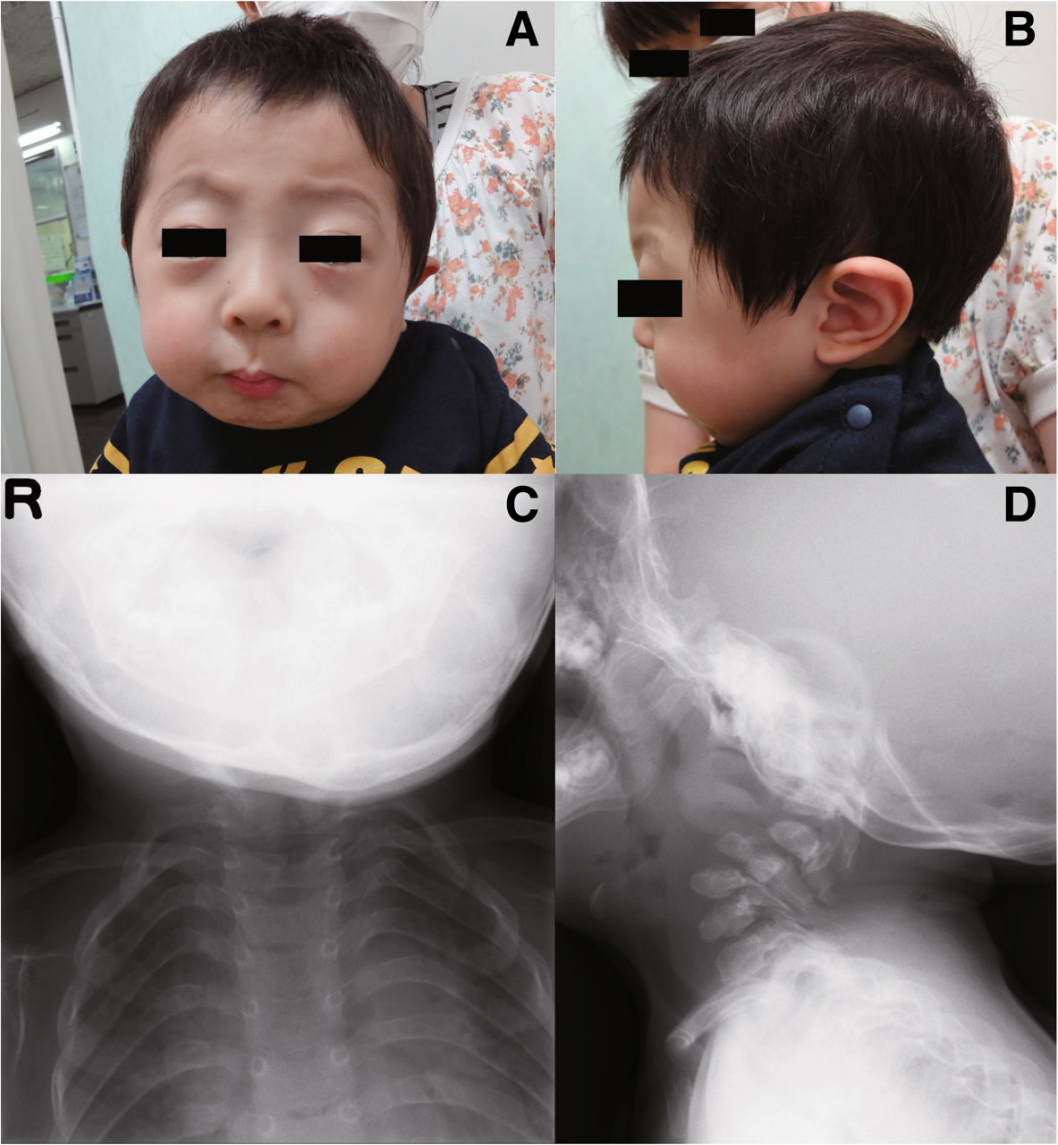
Facial photographs and X-ray films of the patient. **a**, **b** The facial photographs show the typical facial features associated with Schwartz–Jampel syndrome. Fixed facial expression, micrognathia, and low-set ears. **c**, **d** X-ray films show cervical kyphoscoliosis

Preanesthetic examination of the patient showed that he had the typical facial features associated with SJS, including a fixed facial expression with pursed lips, blepharophimosis, micrognathia, and low-set ears (Fig. 2a, b). His X-ray films revealed cervical kyphoscoliosis (Fig. 2c, d). Although his serum creatinine kinase level was slightly elevated (564 U/L), other investigations, including an electrocardiogram, were within the normal range. Because the patient did not show any evidence of airway obstruction, we considered that it was easy to ventilate the patient’s lungs on a mask. Therefore, we planned to induce anesthesia in the patient. We decided to avoid volatile anesthetic agents, which were reported to cause a high temperature in a patient with SJS [3].

Before the operation, the patient was fasted for 6 h with intravenous hydration. Triclofos sodium (1 g) was given orally for anesthetic premedication. Equipment was prepared for a difficult intubation, and two anesthesiologists were present in the operation room. When the patient was brought to the operating theater, pulse oximetry, electrocardiography, and noninvasive blood pressure monitoring were established. General anesthesia was induced with midazolam (1 mg) and fentanyl (10 μg). Mask ventilation was successful. Rocuronium (6 mg) was administered to facilitate tracheal intubation. Direct laryngoscopy was impossible because of the patient’s limited mouth opening. The vocal cords were easily visualized by using an MVS handle with a stylet scope outer diameter (O.D.) 2.5 mm (Additional file 1: Video S1). The device was borrowed from MPI. A 4.0-mm cuffed tracheal tube was inserted, and the cuff inflated to a pressure of 20 cm H_2_O. Anesthesia was maintained by intravenously administering propofol (8–10 mg/kg/h) and remifentanil (0.2–0.5 μg/kg/min) and intermittent doses of rocuronium and fentanyl. At the end of the procedure, residual neuromuscular block was antagonized with sugammadex (18 mg). For postoperative analgesia, 0.2% ropivacaine (3 mL) was infiltrated locally. Tracheal extubation was performed after the patient resumed spontaneous breathing. His postoperative course was uneventful, and he was discharged on postoperative day 2.


Additional file 1: Video S1. Tracheal intubation using the MVS video monitor handle with the stylet scope. Video 1 shows the view of the MVS, which includes the insertion of the stylet scope into the patient’s mouth and trachea. (WMV 13537 kb)


### Discussion

This case report describes the usefulness of the MVS in managing difficult airways. Managing difficult airway associated with limited mouth opening in pediatric patients requires specific skills. Performing an awake intubation is rarely a choice for managing the airway in pediatric patients [4]. We have no choice but manage the airway after induction of general anesthesia in children with anticipated difficult airway. An optic stylet is a suitable option for tracheal intubation in children with restricted mouth opening [5]. There are several optic stylets, which can be used in pediatric patients. Other than the MVS, the Bonfils Retromolar Intubation Fiberscope (Karl Storz GmbH, Tuttlingen, Germany), the Shikani stylet (Clarus Medical, Minneapolis, MN), and the Fiberlightview (Alero, Chino, CA) are available. Only the operator of the intubation can see the airway in these three devices, although they can be connected to a video monitor. The Bonfils Retromolar Intubation Fiberscope is the only optic stylet, which has been evaluated by clinical trials in pediatric patients [6, 7]. The Bonfils fiberscope provided faster intubation than fiberoptic intubation in the clinical nonrandomized study of the pediatric difficult airways [7]. Several case reports, which describe the successful difficult airway management in pediatric patients by using the Shikani stylet, have been published [5, 8, 9]. There are no published articles regarding the usefulness of the Fiberlightview. A conventional stylet scope was reported to be better than a direct laryngoscope for tracheal intubation in patients with simulated cervical spine immobilization [10]. However, a conventional stylet scope cannot be used in pediatric patients. The MVS offers shared vision of the airway during tracheal intubation with the video monitor handle. In contrast to the conventional stylet scope which has a flexible tip, the MVS system has a fixed tip of the stylet scope. Thus, the curve of the tip of the stylet scope used in the MVS cannot be adjusted to each patient. The MVS may not be able to visualize the vocal cords in the situations in which blood or secretion is present in the airway.

## Conclusions

We conclude that an MVS handle with a stylet scope is useful for managing the difficult airways of pediatric patients with SJS.

## Abbreviations

MVS: MultiViewScope
O.D.: Outer diameter
SJS: Schwartz–Jampel syndrome
